# Identifying observable medication use time in administrative databases: a tutorial using nursing home residents

**DOI:** 10.1093/aje/kwaf227

**Published:** 2026-02-05

**Authors:** Daniel A. Harris, Adam D’Amico, Hemalkumar B. Mehta, Lori A. Daiello, Sarah D. Berry, Charles E. Leonard, Yu-Chia Hsu, Douglas Kiel, Kaleen N. Hayes, Melissa Riester, Jimmie E. Roberts, Laura Reich, Peyton Free, Andrew R. Zullo

**Affiliations:** 1 Department of Epidemiology, College of Health Sciences, University of Delaware, Newark, DE, United States; 2 Department of Health Services, Policy, and Practice, School of Public Health, Brown University, Providence, RI, United States; 3 Center for Gerontology and Healthcare Research,School of Public Health, Brown University, Providence, RI, United States; 4 Center for Drug Safety and Effectiveness, Johns Hopkins Bloomberg School of Public Health, Johns Hopkins University, Baltimore, MD, United States; 5 Department of Epidemiology, Johns Hopkins Bloomberg School of Public Health, Johns Hopkins University, Baltimore, MD, United States; 6 Hinda and Arthur Marcus Institute for Aging Research, Hebrew Senior Life, Boston, MA, United States; 7 Department of Medicine, Beth Israel Deaconess Medical Center and Harvard Medical School, Boston, MA, United States; 8 Department of Biostatistics, Epidemiology, and Informatics, Perelman School of Medicine, University of Pennsylvania, Philadelphia, PA, United States; 9 Department of Epidemiology, School of Public Health, Brown University, Providence, RI, United States

**Keywords:** pharmacoepidemiology, aging, Medicare, bias, epidemiologic methods

## Abstract

Nursing home (NH) residents are an important population for pharmacoepidemiologic research due to their prevalence of multimorbidity and polypharmacy. Medicare claims are commonly used to study medication use in this population, but medications dispensed during hospitalizations or post-acute care are unobservable due to bundled payment structures. We developed algorithms to identify NH days when medication dispensings can be observed in claims. Using a cohort of NH residents in the United States from 2013 to 2020, we linked Medicare fee-for-service (FFS) claims with Minimum Data Set clinical assessments. NH days were classified as “observable medication use time” if residents were enrolled in Medicare parts A, B, and D were not receiving post-acute care and were not hospitalized. Among 12.3 million NH residents and 2.7 billion NH days, 1.1 billion days (72.4% of Medicare-enrolled days and 39.6% of all NH days) were identified as observable medication use time. Within the first 100 days of NH admission, 27.3% of days were medication-observable, increasing to 89.4% after 100 days. On average, we identified 68% more person-time, and 51% more residents, compared to standard 100-day definitions for “long-stay” NH residents. Our algorithms enhance researchers’ ability to measure medication exposure time, improving the validity of pharmacoepidemiologic studies.

## Introduction

Medication use among nursing home (NH) residents has been a focus of clinical, epidemiologic, and health services research for decades.^1–4^ NH residents are a priority population for pharmacoepidemiologic research due to their advanced age and high burden of multimorbidity and polypharmacy.^5^ Medication use is widespread in the NH, with residents taking between 4 and 17 medications on average,^6^ thereby increasing the risk of drug interactions and polypharmacy-associated morbidity.^7,8^ However, evidence on medication safety and effectiveness is limited in this population because clinical trials seldom include NH residents. As a result, pharmacoepidemiologic studies evaluating the use, safety, and effectiveness of medications in NHs are essential to generate clinical evidence.

Health administrative data, particularly Medicare claims linked to Minimum Data Set (MDS) clinical assessments, are commonly used to study medication use and outcomes in NH residents.^2,3,5^ However, a limitation of Medicare claims is that medications dispensed during certain periods—such as hospitalizations and post-acute skilled nursing care—are unobservable due to bundled payment structures. This presents a major challenge for accurately measuring medications and for defining person-time denominators in pharmacoepidemiologic research.^9,10^

Most NHs in the United States provide 2 types of care: shorter term care following a hospitalization or illness (ie, “skilled nursing” or “short-stay” NH residents) and longer term custodial care (ie, “long-stay” NH residents).^11^ Medicare part A currently covers up to 100 days of skilled nursing care, and long-term care is typically paid for by Medicaid, private insurance, or out-of-pocket funds. Medications dispensed during part A-covered skilled nursing care are not individually billed, making them unobservable in Medicare claims. Prior studies have used different approaches to classify short-stay vs long-stay NH residents to measure medication exposure, but these methods dichotomize individuals into 2 groups based on length-of-stay cut-offs (eg, restricting to residents with >100 days length of stay to identify long-stay NH residents and medication exposures).

To support future pharmacoepidemiologic research among US NH residents, we developed an algorithm to identify periods of observable medication use time using Medicare claims. Our method builds upon prior approaches by shifting from resident-level classifications to person-time–level classifications of medication observability. This tutorial describes our algorithm and provides publicly available SAS code with techniques to improve efficiency when working with large administrative datasets (ie, parallel processing code).

## Methods

### Study design and data sources

We conducted a cohort study using Medicare part A (Medicare Provider Analysis and Review [MedPAR]), part B, and part D files linked to MDS clinical assessments. All datasets were 100% files and included all individuals who spent at least 1 day in the NH between January 1, 2013, and December 31, 2020. Medicare part A includes information on inpatient hospitalizations, emergency department visits, and post-acute skilled nursing care; Medicare part B includes information on outpatient professional services and visits; and Medicare part D includes information on prescription medication–dispensing claims. NH MDS assessments capture a wide range of clinical information upon admission to the NH, at quarterly intervals following admission, and if there is a significant change in a resident’s health status. Strict rules govern when MDS assessments should be completed, and they are submitted to the Centers for Medicare & Medicaid Services (CMS) for evaluation, quality reporting, licensure, and payment for eligible clinical services.^12^ Finally, the Medicare Beneficiary Summary File (MBSF) was used to determine Medicare enrollment status.

All data were linked using an anonymized Medicare beneficiary ID provided through a data use agreement between Brown University and the CMS Research Data Assistance Center.^13^ This study was considered exempt by the Brown University and University of Delaware Institutional Review Boards given its use of secondary datasets.

### Study population

Our study population included US NH residents enrolled in Medicare fee-for-service (FFS, ie, parts A and B). Residents enrolled in a Medicare Advantage plan were excluded due to the limited completeness of the encounter data during the study period.^14^ Medicare enrollment status was determined as of each day in the NH using the MBSF (a process described below in step 2). No other eligibility criteria were applied for the purposes of the tutorial.

### Step 1: identifying nursing home time

**Figure 1.**
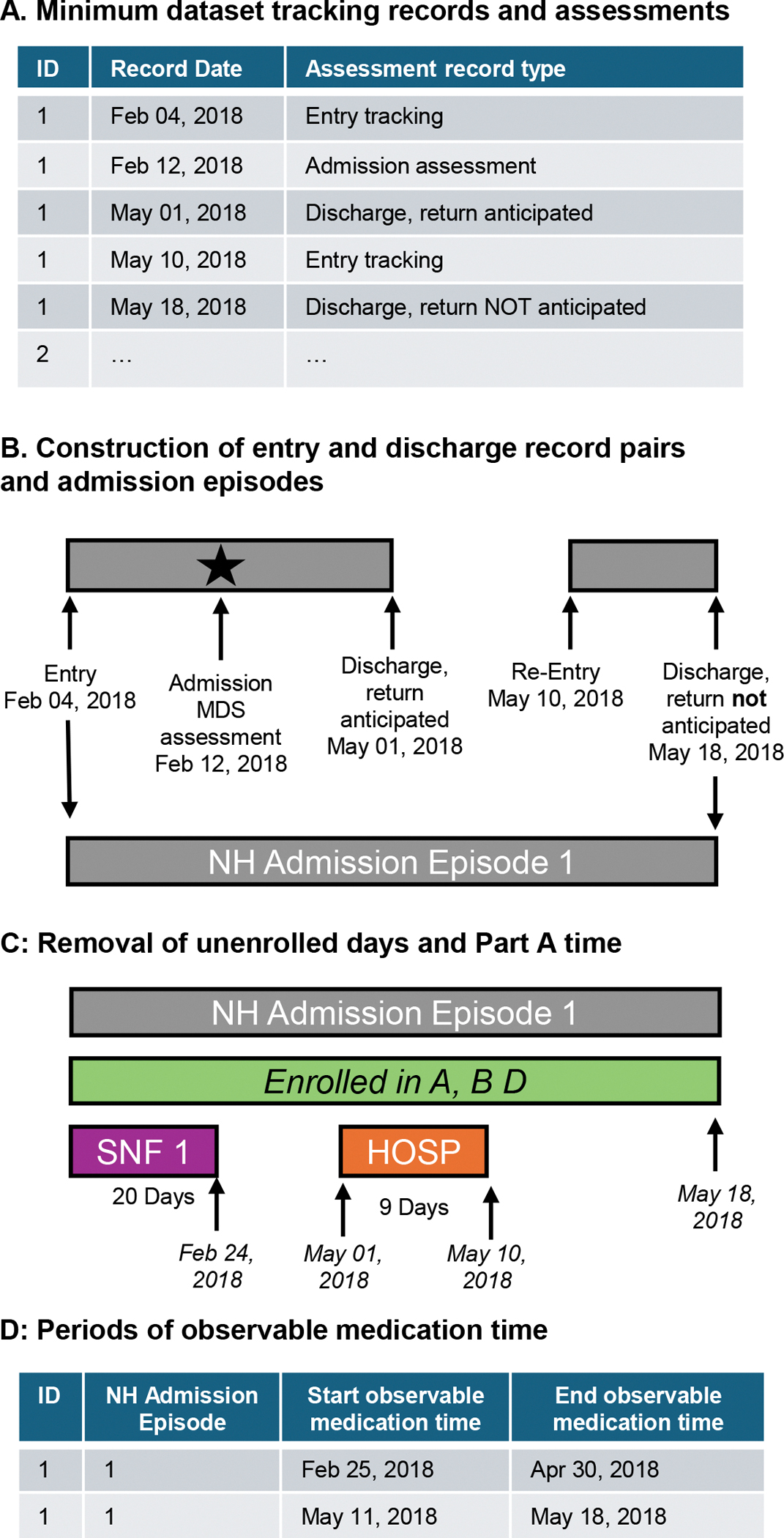
Schematic of data processing steps to identify observable medication use time in NH residents. Figure is not drawn to scale; SNF, post-acute skilled nursing care; HOSP, inpatient hospitalization; NH, nursing home; MDS, Minimum Data Set.

Goodwin et al. and Intrator et al. developed methods for identifying NH time and classifying short- vs long-stay residents, whereas our algorithm identifies NH time during which medications are observable vs unobservable for pharmacoepidemiologic research.^15,16^ Similar to other investigators,^4,15^ we used the MDS database as the foundation of our cohort-generating process. We anchored our cohort on the MDS because these assessments are required for federal certification, occur regularly in NHs, and are completed by trained nursing staff. The MDS data structure should be oriented in “long” or “person-period” format, such that individual residents will have multiple rows in the dataset representing different MDS tracking records (eg, entry and discharge records) and assessments (eg, admission assessment; Figure 1A) over time.

We distinguish between 2 types of NH time based on the MDS tracking records and MDS assessments: (1) “entry-anchored” NH episodes and (2) “admission assessment–anchored” NH episodes (Figure S1). Entry-anchored NH episodes begin with an MDS entry date to the NH and end upon a subsequent discharge date (if present). Entry-anchored NH episodes include the maximum amount of MDS-based NH time, though these episodes may represent periods of time that are far removed from any clear MDS admission (see Table S1 for a summary of total NH time for “entry-anchored” episodes). Depending on the goals of the study, investigators may choose to require NH episodes to include an MDS admission assessment as a strategy to define a natural study index date, to identify a clinically meaningful start to a NH episode, and to capture the admission’s associated clinical information. To simplify the methods being described and reporting of results, we focus on “admission assessment–anchored” NH episodes unless otherwise specified (Figure 1B). Admission assessment–anchored NH episodes end upon the occurrence of either:

1. An MDS discharge record and no return to the NH was anticipated (ie, MDS 3.0 field A0310F = 10 or 12); or
2. An MDS discharge record and a return was anticipated (ie, MDS 3.0 A0310F = 11) but there was no subsequent reentry into the NH within 30 days (per the MDS manual and guidance from CMS, a return beyond 30 days triggers a new admission vs reentry).^12^

During the processing of the MDS data, we further encountered and made decisions for the following conditions:

1. There may be distinct NH episodes that overlap each other in the administrative data (see Figure S1 for an example schematic). We combined these records into 1 NH record. All NH episodes were sorted by their entry and discharge dates and evaluated sequentially. Episodes were considered overlapping if the entry date for 1 episode occurred on or before a previously documented discharge date. We used the first entry date and last discharge date to combine overlapping NH episodes.
2. If an NH entry had no accompanying discharge record, we assumed that the resident was still in the NH, and we set the end date to the last day of the study period.
3. All episodes were truncated at the date of death from the Medicare records, if applicable.

For the following steps, we expanded the final admission assessment–anchored NH episodes to the day level, such that every row represented a unique day in the NH per resident and per NH admission (as residents could have multiple NH admissions over the study period). For the purposes of this study, we define “observable medication use time” as days in the NH when medications could be measured using part D claims.

### Step 2: flagging unobservable medication time due to lack of enrollment

As described above, medications dispensed to NH residents are only observable in the health administrative data if the resident was enrolled in Medicare part D. In the absence of Medicare Advantage data, we further required residents to be enrolled in Medicare FFS to measure clinical characteristics. Step 2 uses the MBSF database to flag NH days during which residents were unenrolled in Medicare parts A, B, and D. For each NH day, a binary flag variable indicated whether the resident met the enrollment criteria.

### Step 3: flagging unobservable medication time due to post-acute skilled nursing care

During a single NH episode, it is common for residents to enter the NH receiving post-acute skilled nursing care and then transition to long-term NH care or discharge back to the community. Additionally, residents can have several discrete periods of post-acute skilled nursing care while in the NH. Medications dispensed during these periods are unobservable due to bundled payments under Medicare part A. We used MedPAR to identify the start and end of post-acute skilled nursing care to flag NH days covered under part A. We used binary variables to indicate whether a given day coincided with part A payments for post-acute skilled nursing care.

### Step 4: flagging unobservable medication time due to inpatient hospital stays

Like post-acute skilled nursing care, medications dispensed during inpatient hospitalizations are unobservable. We used MedPAR to identify the start and end dates of inpatient hospitalizations. NH days that coincided with inpatient hospitalizations were flagged using a binary variable.

### Step 5: removing unobservable medication days and constructing new NH episodes

In the final day-level database, indicator variables captured whether a given day was enrolled in Medicare parts A, B, and D (step 2); billed as post-acute skilled nursing care (step 3); or spent in the hospital as an inpatient (step 4). All NH days that were unenrolled, billed to SNF care, or spent in the hospital were removed. New and continuous periods of “observable medication use” time in the NH were generated and stored as new rows, with each row representing the start and end of a distinct period of observable medication use time (Figure 1C and D).

We include all variables and variable descriptions in the final dataset that are produced by our algorithm in an online repository (https://doi.org/10.5281/zenodo.15012812). Due to the size of the day-level datasets, we include publicly available code to conduct parallel processing in SAS. Parallel processing is a coding technique that opens several SAS programs at the same time (ie, ~10 SAS programs). Each SAS program processes a different “partition” of the overall database, which has been divided into smaller samples. For example, the overall database of all NH days was divided into 100, 1% datasets (ie, dataset_1_p to dataset_100_p). The parallel processing code instructs each of the 10 SAS programs to process a different set of datasets. For example, SAS program 1 processes Dataset_1_p to Dataset_10_p, while SAS program 2 processes Dataset_11_p to Dataset_20_p. Once all datasets are processed, they are appended to generate 1 final analytic dataset. Finally, we include a synthetic dataset to show the data structure once all processing is complete.

### Covariates

To describe our NH cohort and demonstrate how its demographic composition changes following the removal of unobservable medication use time, we measured residents’ sociodemographic (eg, age and sex) and select chronic conditions (eg, diabetes and hypertension) as of residents’ first NH admission assessment using the MDS.

### Analyses and software

We used descriptive statistics (eg, proportions, means, and SDs) to describe the NH population prior to and following unobservable medication use time removal. We also summarized the number of observable medication use days following each data processing step by periods of time following admission to the NH (ie, 1–30 days; 31–60 days; 61–100 days; and 101+ days in the NH). Missing data was uncommon, but some discharge dates in MedPAR were missing. We imputed missing MedPAR discharge dates using the length-of-stay variable.

To demonstrate differences in our algorithm to approaches commonly employed in NH pharmacoepidemiology studies, we compared the total number of NH days, total number of NH residents, and gabapentin medication prevalence identified by our entry- and admission assessment–anchored algorithms to the standard 101-day definition for long-stay NH residents outlined in Goodwin et al.’s “method 2” algorithm.^15^ All analyses were conducted in SAS version 9.4 (Carey, NC).

## Results

### Admissions, length of stay, and person-time

We identified a total of 12 336 061 unique NH residents, 18 535 715 NH admissions, and 2 685 903 261 NH days (7 353 602 NH years) from 2013 to 2020 (Table 1). Before removing unobservable medication use days, the mean length of stay was 144.9 days (SD = 348.6 days), and the median was 31 days (IQR = 20–70 days). NH residents had 1.5 (SD = 1.0) admission episodes, on average, during the study period.

### Proportion of NH days with observable medication use time

**Figure 2.**
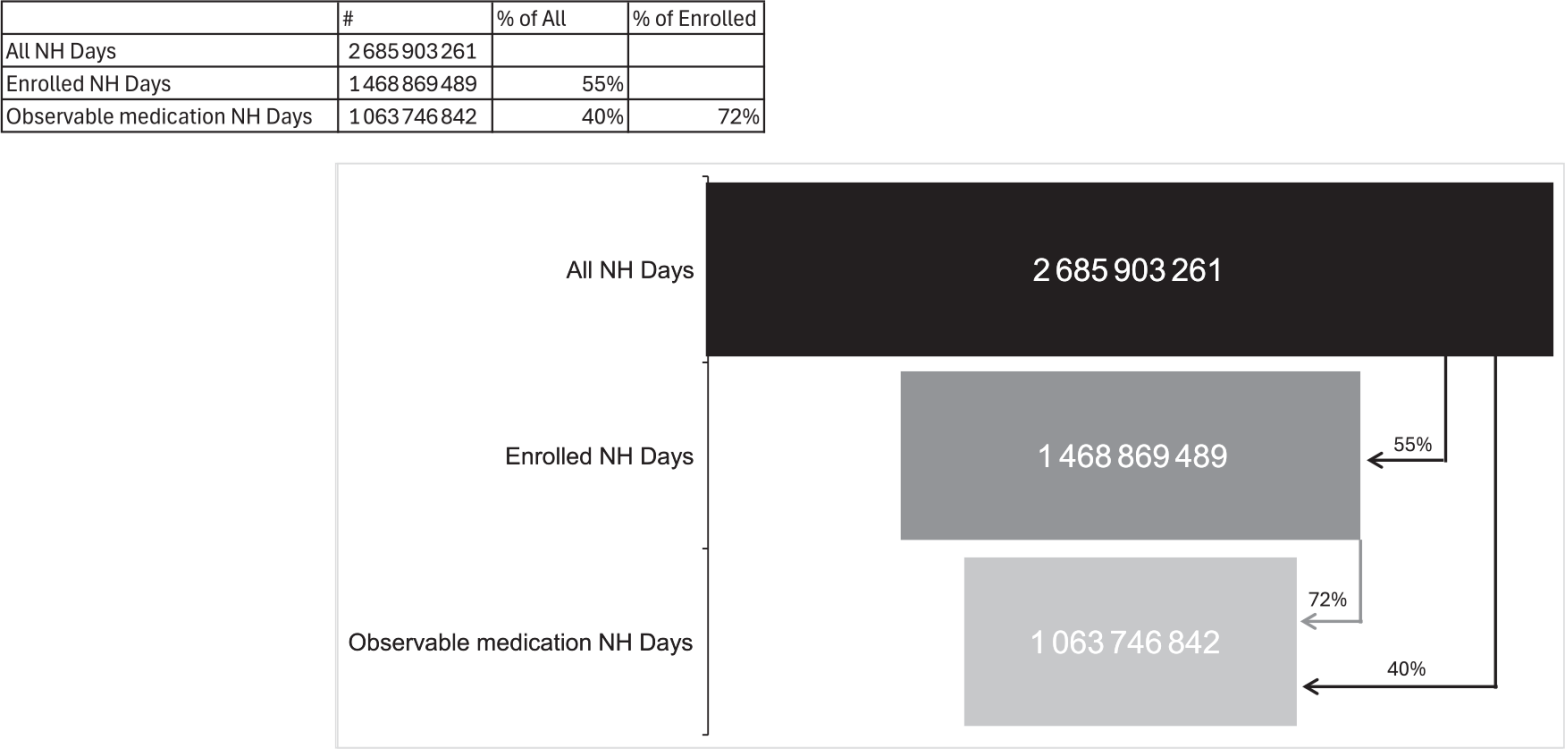
Total number of NH days prior to and following the removal of unobservable medication use time. Enrolled NH days represent days when the NH resident was enrolled in Medicare FFS (parts A and B) and Medicare part D. Observable medication NH days represent days when residents were enrolled in Medicare FFS, part D, not receiving post-acute skilled nursing care, and not in the hospital as an inpatient. NH, nursing home; FFS, fee-for-service.

A total of 1 217 033 772 days were removed due to a lack of enrollment in Medicare parts A, B, and D (45.3% of total NH days; Table 1). Of the remaining 1 468 869 489 days, 386 022 155 (26.3%) and 29 012 885 (2.0%) were removed due to post-acute skilled nursing care or being in the hospital, respectively. A total of 1 063 746 842 days remained, representing days when medication use could be observed in the NH (72.4% of Medicare-enrolled NH days and 39.6% of all NH days). Figure 2 visualizes the reduction in all nursing home time after Medicare enrollment and periods of part A coverage were removed.

### Medication observability over time since NH admission

Within the first 30 days of NH admission, there were 28 938 337 observable medication days (14.1% of all Medicare-enrolled days in the NH during this period). The proportion of observable medication use days increased according to time following NH admission: 31–60 days after admission = 32 781 689 observable days (31.0% of Medicare-enrolled days during this period); 61–100 days after admission = 48 040 893 observable days (52.4% of Medicare-enrolled days during this period); and 101+ days after admission = 953 985 923 observable days (89.4% of Medicare-enrolled days during this period). Within the first 100 days of NH admission, a total of 109 760 919 days, or 27.3% of Medicare-enrolled days, represented observable medication use time in the NH.

### Differences in resident characteristics by medication observability

Table 2 compares the NH resident characteristics prior to and following the removal of unobservable medication use days. Although removing unobservable medication use days greatly reduced the total number of NH residents (initial cohort, *n* = 12 336 061 vs. cohort with at least 1 observable medication day and enrolled in Medicare FFS, *n* = 2 617 363), there were relatively small differences in the demographic characteristics between cohorts. In the initial cohort, the average age was 76.2 years (SD = 13.0), 59.2% were women, and 77% were White race. In the FFS-enrolled cohort with at least 1 day of observable medication use time, the average age was 79.0 (SD = 12.1), 63.2% were women, and 78.1% were White race. The mean length of observable medication use time was 255.8 days (SD = 354.7 days), with a median length of 76 days (IQR = 19–272).

### Comparison to the standard 100-day definition for long-stay NH residents

**Figure 3.**
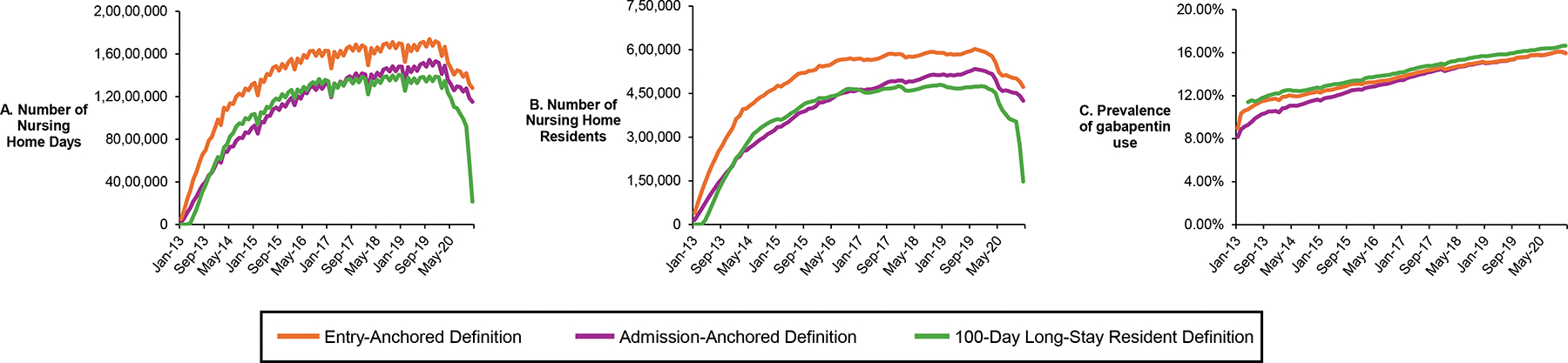
Comparison of the entry- and admission-anchored observable medication time algorithms to the standard 100-day definition of long-stay NH residents in terms of total number of NH days (A), total number of unique NH residents (B), and prevalence of gabapentin (C) over time. NH, nursing home.

In January 2017, our entry-anchored algorithm identified 569 752 unique NH residents and 16 266 413 NH days; our admission assessment–anchored algorithm identified 463 284 unique NH residents and 13 200 548 NH days; and the standard 100-day algorithm for identifying “long-stay” NH residents identified 460 695 NH residents and 13 426 698 NH days (Table S2). Over time, our entry-anchored algorithm identified 68% more NH time, and 51% more unique NH residents, compared to the standard 100-day definition for long-stay NH residents (Figure 3). Among residents identified using the 100-day algorithm, 8.5% of their NH days (over 87 200 000 days) had unobservable medication use time (Table S3). Residents identified in the 100-day long-stay algorithm had a similar average age to those identified in our algorithms, but they had a greater prevalence of dementia. Gabapentin prevalence was similar, though slightly lower in our algorithms.

## Discussion

We developed an algorithm to identify observable medication use time among US NH residents using Medicare FFS claims linked to MDS assessments. This approach addresses a challenge in NH pharmacoepidemiologic research: medications dispensed during hospitalizations and post-acute skilled nursing care are unobservable in Medicare part D claims due to bundled payment structures under Medicare part A. Our findings demonstrated that a substantial proportion of NH days were unobservable, particularly shortly after NH admission, when many residents receive post-acute skilled nursing care. By identifying NH days when medication dispensings can be reliably observed, our method allows researchers to define at-risk person-time more accurately, improve exposure measurement, and reduce immeasurable time biases in studies reliant on Medicare claims. Unlike prior approaches that categorized residents as short vs long stay, our algorithm captures dynamic transitions in care settings at the person-day level, providing a more precise measure of medication exposure time. These advancements have broad implications for improving the validity of medication use research in NH populations, especially for capturing medication and vaccine exposures shortly after NH admission. We also note that they can be adapted to other databases where bundled payment models affect medication observability.

CMS uses 101 days in the NH to distinguish between short- and long-stay NH residents when generating publicly reported quality indicators and broadly distinguishing between different types of residents (ie, those receiving post-acute skilled nursing care vs those living in the NH as their primary residence). This cut-off is informed by the payment schedule for post-acute skilled nursing care. Medicare part A will cover 100% of skilled nursing care for the first 20 days and 80% for days 21–100.^15^ As part A will cover up to the first 100 days of skilled nursing care, NH drug studies commonly condition on having 90–101 or more days in the NH as an inclusion criteria to blanketly avoid immeasurable time.^17–19^ However, a minority of residents require the full 100 days and NH residents can transition in and out of Medicare part A coverage even after they have lived in the facility for more than 100 days (ie, due to hospitalizations and procedures that occur during their NH tenure).^20–22^ NH researchers have recognized the potential for the 101-day cut-off to misclassify the types of NH residents, and tested different cut-offs (eg, 30 days).^15,16,23^ However, these approaches still focus on classifying NH residents vs person-time, and without the additional steps outlined in our algorithm, may misclassify medication observability. For example, even among long-stay NH residents identified using Goodwin et al.’s approach of 101 or more days in the NH, over 8% of NH time was medication-unobservable. We also note that our algorithms had large differences in person-time likely due to the decision to end on an MDS assessment, whereas our algorithms end on the last day of the study period end date if there was no discharge record. Nonetheless, the algorithms created by Goodwin et al. and others serve an important, yet different, purpose; one that is useful for distinguishing between NH residents with different care needs. We argue that our algorithm is better suited for pharmacoepidemiologic studies of medication use, safety and effectiveness, where a more continuous evaluation of person-time is preferred to binary cut-offs based on length of stay.

Similar to our algorithm, Wei et al. used a combination of US health administrative databases to process NH time at the day level to obtain precise lengths of stay in the facility and to more accurately apply CMS’ 101-day definition of “long-stay” NH residents.^23^ Intrator et al.^16^ developed the “residential history file” to track individuals through several types of care over time, including post-acute care, home health, NH, hospice, emergency department, and hospital care. While these methods have been adapted to pharmacoepidemiologic studies,^24^ they were not designed to identify observable medication use time in the NH, leading to potential methodological variation across studies. Using our method, we observed a substantial number of days shortly after NH admission whereby medications could be observed in part D claims—information that may otherwise be discarded if traditional short- vs long-stay NH classifications were used and based on “hard” cut-offs.

In 2008, Suissa described “immeasurable time bias” in pharmacoepidemiologic database studies as an important threat to internal validity.^10^ For example, in a case-control study, a greater number or length of prior hospitalizations among the cases will ostensibly result in lower medication exposure rates among the cases due to this time being unobservable. Suissa demonstrated that the association between drug exposure and mortality varied widely in the presence of immeasurable time—from a 40% reduction in mortality rate to a 35% increased mortality rate.^10^ Recently, authors have recommended controlling for immeasurable time periods (information that is quantified in our algorithm at the day level) as a time-varying covariate.^25^ Our algorithm could be used to identify and balance day-level changes in person-time when exposure to medications (or vaccinations) are immeasurable.

**Figure 4.**
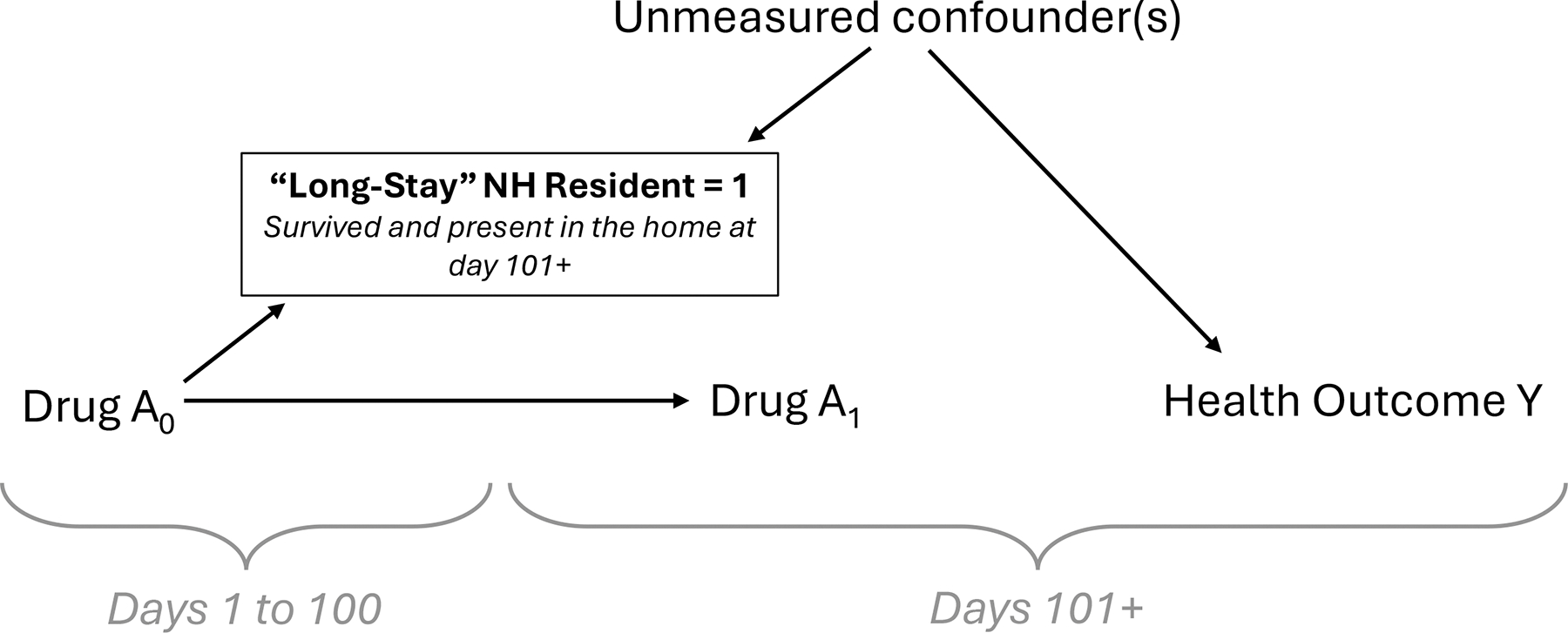
Directed acyclic graph depicting collider stratification by conditioning on long-stay NH status when assessing the association between exposure drug A_1_ and health outcome Y. NH, nursing home.

It can also be noted that conditioning on “long-stay” NH status may result in selection bias and/or immortal time bias when examining a putative causal relationship between drug A and health outcome Y (Figure 4). Due to bundled payments, drug A will not be observable until after an NH resident transitions from short to long stay. Under conventional wisdom, investigators may restrict to residents who have survived and remained in the home for 101 or more days, thereby estimating the relationship between drug A_1_ and health outcome Y. In this simplified directed acyclic graph (DAG), long-stay NH status is a common effect of drug A_0_ administered during the first 100 days and unmeasured confounder U. Conditioning on long-stay status introduces collider stratification along the path from drug A_1_ ← drug A_0_ → [long-stay NH status] ← U → Y, resulting in a biased estimate of the average causal effect of drug A_1_ on health outcome Y. Our algorithm does not make assumptions about medication observability during the first 100 days of a resident’s NH stay and enables investigators to potentially balance unobservable time. Under relevant assumptions, biasing paths could also be closed by adjusting for A_0_ conditional on those with observable time.^26^ Although the DAG depicted here is a simplified version of real-world causal structures as an illustrative example, it depicts how historically common practices in NH pharmacoepidemiology can introduce selection bias and immortal time bias, and how DAGs with selection nodes can be useful for obtaining unbiased causal estimands in the presence of complex missing data patterns.

There are several limitations of this method. First, Medicare claims are only able to capture prescription medications, so nonprescription medications, supplements, prescriptions paid out-of-pocket, and Veterans Affairs–paid prescriptions remain unobservable in the absence of other data sources. Finally, the initial cohort size was large, and the requirement for enrollment in Medicare FFS resulted in a large loss of individuals. Therefore, results may not fully generalize to NH residents with Medicare Advantage or those with partial coverage (ie, enrolled in Medicare part A only). As the quality of the Medicare Advantage encounter data improves, future investigators might consider incorporating Medicare Advantage beneficiaries into their studies. Strengths of the current study include its use of several linked health administrative databases to identify unobservable medication time among a national sample of US NH residents. Future research should quantify the extent of selection and immortal time biases that can be introduced through binary cut-offs of short- vs long-stay residents when evaluating drug and vaccine effects, including how such biases can be mitigated through our day-level assessment of medication observability.

## Conclusions

In a cohort study of 12 million NH residents over nearly 1 decade of time, we used a combination of Medicare enrollment, Medicare claims, and MDS assessment data to develop an algorithm that identifies periods of observable medication use time in NHs. As opposed to classifying residents as short vs long stay, our approach classifies person-time as observable vs unobservable for medication use, thereby enhancing researchers’ ability to precisely measure medication exposure time and reduce potential information and selection biases in NH pharmacoepidemiologic research. We also found a notable proportion of observable medication use time shortly after admission to the NH—time that may otherwise be discarded if the standard 101-day cut-off used by CMS was applied. This algorithm can be used in combination with existing approaches for handling immeasurable person-time and to maximize the amount of available medication-related exposure information among NH residents.^10,25,27^

## Supplementary Material

Supplementary Materials

Supplementary material

Supplementary material is available at the *American Journal of Epidemiology* online.

## Figures and Tables

**Table 1.** Reductions in nursing home time at each stage in the algorithm to identify nursing home days with observable medication use time in Medicare claims among residents of US nursing homes between 2013 and 2020.

	Days 1–30	Days 31–60	Days 61–100	Days 101+	All days

Step 1: Prior to person-time removal					
Number of unique residents	12 336 061	7 271 139	4 556 953	3 317 609	12 336 061
Number of nursing home admission episodes	18 535 715	9 394 124	5 197 752	3 570 436	18 535 715
Total number of days	459 832 789	207 197 264	171 031 431	1 847 841 777	2 685 903 261
Mean number of days (SD)	24.8 (6.6)	22.1 (10.5)	32.9 (12.4)	517.5 (593.7)	144.9 (348.6)
Median number days (IQR)	30.0 (20.0–30.0)	30.0 (13.0–30.0)	40.0 (29.0–40.0)	285.0 (70.0–768.0)	31.0 (20.0–70.0)
Step 2: Medicare enrollment					
Total number of days unenrolled in part A, B, D	254 994 642	101 428 163	79 405 849	781 205 118	1 217 033 772
Total number of days enrolled in part A, B, D	204 838 147	105 769 101	91 625 582	1 066 636 659	1 468 869 489
Step 3: Time in skilled nursing care^a,b^					
Total number of enrolled days in SNF	172 972 223	70 176 713	41 584 448	101 288 771	386 022 155
Total number of enrolled days not in SNF	31 865 924	35 592 388	50 041 134	965 347 888	1 082 847 334
Step 4: Time in hospital^a,b^					
Total number of enrolled days in the hospital	10 300 179	3 557 164	2 431 045	12 724 497	29 012 885
Total number of enrolled days not in the hospital	194 537 968	102 211 937	89 194 537	1 053 912 162	1 439 856 604
Step 5: Observable drug time					
Total observable medication days	28 938 337	32 781 689	48 040 893	953 985 923	1063 746 842
Observable drug time among all enrolled days (%)	14.1%	31.0%	52.4%	89.4%	72.4%
Number of residents with at least 1 day of observable drug time	1 275 260	1 431 592	1 478 432	1 861 440	2 617 363
Number of nursing home admission episodes with at least 1 day of observable drug time	1 410 776	1 547 353	1 570 744	1 961 682	2 945 832
Number of unique observable drug time episodes	1 434 351	1 585 433	1 629 173	3 411 082	4 711 549

Abbreviation: SNF, post-acute skilled nursing care. All results were derived from the admission assessment-anchored algorithm.

a Calculated among the days enrolled in Medicare parts A, B, and D.

b Adding (+) skilled nursing and hospitalization days may exceed the difference of enrolled days due to overlapping/double counting of days. In some cases, a single day can be attributed to skilled nursing care and a hospitalization.

**Table 2.** Sociodemographic characteristics, nursing home length of stay, and nursing home admissions prior to and following the removal of unobservable medication use time among US nursing home residents between 2013 and 2020.

	Initial cohort identified in the MDS database	Eligible^a^ cohort with at least 1 day of observable medication time in the NH

Number of unique residents admitted during the study	12 336 061	2 617 363
Demographic characteristics		
Age in years at earliest observed admission (mean [SD])	76.2 (13.0)	79.0 (12.1)
Sex		
Male (%)	5 036 243 (40.8)	964 281 (36.8)
Female (%)	7 299 785 (59.2)	1 653 080 (63.2)
Race and ethnicity^b^		
Black (%)	1 382 227 (11.2)	294 234 (11.2)
Asian (%)	238 182 (1.9)	47 122 (1.8)
Hispanic (%)	608 529 (4.9)	118 686 (4.5)
White (%)	9 492 853 (77.0)	2 044 821 (78.1)
American Indian or Alaska Native (%)	52 014 (0.4)	11 548 (0.4)
Native Hawaiian or Other Pacific Islander (%)	45 322 (0.4)	7634 (0.3)
Other race or ethnicity (%)	541 638 (4.4)	98 057 (3.8)
Missing	17 467 (0.1)	2436 (0.1)
Nursing home stay characteristics		
Average number of nursing home admissions per resident, mean (SD)	1.5 (1.0)	1.74 (1.2)
Average length of stay in the nursing home in days, per admission, mean (SD)	144.9 (348.6)	344.1 (531.3)
Average length of observable drug time episode (SD)	NA	255.8 (354.7)
Median length of observable drug time episode (IQR)	NA	76 (19–272)
US region at earliest observed admission^c^		
Northeast (%)	2 622 132 (21.3)	572 246 (21.9)
Midwest (%)	3 169 356 (25.7)	756 142 (28.9)
South (%)	4 285 346 (34.7)	950 853 (36.3)
West (%)	2 248 170 (18.2)	338 077 (12.9)

Abbreviations: FFS, fee-for-service; NA, not applicable.

a Individuals enrolled in Medicare FFS.

b Race and ethnicity categories were measured using the Minimum Data Set and are not mutually exclusive. Other race or ethnicity is defined as not identifying with the specified categories.

c Region counts and percentages are based on the first admission episode per resident.

## Data Availability

Due to data use agreements and privacy restrictions, study data are not publicly available. To access Medicare claims data, we recommend readers contact the Research Data Assistance Center (ResDAC; CMS contract number: HHSM-500–2015-00558G). To provide a sense of the final cohort, we include a synthetic (fabricated) data that approximates what the data look like for analysis. We also provide all SAS code used in the study in an online repository (see https://doi.org/10.5281/zenodo.15012812).
