# Supplementary material for: Identifying observable medication use time in administrative databases: a tutorial using nursing home residents
Source: Am J Epidemiol. Author manuscript; Available in PMC 2026 Jan 17. (PMC12809372; doi:10.1093/aje/kwaf227)

**SUPPLEMENTARY MATERIALS**

Identifying observable medication use time in administrative databases: A tutorial using nursing home residents

Daniel A. Harris, Adam D’Amico, Hemalkumar B. Mehta, Lori A. Daiello, Sarah D. Berry, Charles E. Leonard, Yu-Chia Hsu, Douglas Kiel, Kaleen N. Hayes, Melissa Riester, Jimmie E. Roberts, Laura Reich, Peyton Free, Andrew R. Zullo

**Table S1.** Number of unique nursing home residents and person days in using the entry-anchored algorithm

**Table S2.** Characteristics of the nursing home residents identified by the entry-anchored, admission-anchored, and standard 100-day definition of long-stay nursing home residents over time

**Table S3.** Description of all nursing home residents identified using the 100-day long-stay definition and their overlap with the entry-anchored algorithm

**Figure S1**. Schematic of overlapping nursing home episodes in the health administrative data

*Additional supplementary material, including all SAS code, parallel processing SAS code, variable descriptions, and a synthetic dataset can be found on the study repository:* [***https://zenodo.org/records/15062201***](https://zenodo.org/records/15062201)***.***

**Table S1.** Number of unique nursing home residents and person days in the nursing home without requiring an admission assessment

| **N unique beneficiaries** | **N unique NH entries** | **N total person days in the NH** | **N total person years in the NH** |
| --- | --- | --- | --- |
| 15,407,554 | 31,679,055 | 3,218,239,301 | 8,811,059 |

**Table S2.** Characteristics of the nursing home residents identified by the entry-anchored, admission-anchored, and standard 101-day definition of long-stay nursing home residents over time

|  | January 2013 | | | January 2017 | | | December 2020 | | |
| --- | --- | --- | --- | --- | --- | --- | --- | --- | --- |
| Characteristics | Entry-  anchored | Admission-anchored | 101-Day Long-Stay Definition ^d^ | Entry-  anchored | Admission-anchored | 101-Day Long-Stay Definition | Entry-  anchored | Admission-anchored | 101-Day Long-Stay Definition |
| N Unique Residents | 39,312 | 15,187 | 0 | 569,752 | 463,284 | 460,695 | 472,365 | 425,119 | 147,492 |
| N Unique Days | 497,977 | 209,810 | 0 | 16,266,413 | 13,200,548 | 13,426,698 | 12,800,932 | 11,512,377 | 2,151,790 |
| N Gabapentin Days | 44,548 | 17,125 | 0 | 2,237,996 | 1,776,717 | 1,902,219 | 2,040,220 | 1,837,641 | 358,266 |
| Age, mean (SD) a | 80.1 (12.6) | 80.4 (12.5) | - | 81.2 (12.2) | 81.5 (12.0) | 81.5 (12.2) | 78.8 (12.5) | 79.1 (12.4) | 79.5 (12.5) |
| Age, median (Q1, Q3) a | 83 (73, 89) | 83 (73,89) | - | 83 (74, 90) | 84 (74, 90) | 84 (74, 91) | 80 (71, 88) | 81 (71, 89) | 81 (71, 89) |
| Age, n (%) ^a^ |  |  |  |  |  |  |  |  |  |
| <65 | 4,596 (11.7) | 1,707 (11.2) | - | 56,234 (9.9) | 43,016 (9.3) | 44,792 (9.7) | 57,513 (12.2) | 49,536 (11.7) | 16,976 (11.5) |
| 65-69 | 2,835 (7.2) | 1,072 (7.1) | - | 40,538 (7.1) | 31,796 (6.9) | 31,668 (6.9) | 48,961 (10.4) | 42,882 (10.1) | 14,373 (9.7) |
| 70-74 | 3,629 (9.2) | 1,296 (8.5) | - | 53,978 (9.5) | 42,858 (9.3) | 42,067 (9.1) | 57,193 (12.1) | 50,260 (11.8) | 16,967 (11.5) |
| 75-79 | 4,712 (12.0) | 1,759 (11.6) | - | 67,169 (11.8) | 54,325 (11.7) | 52,923 (11.5) | 62,290 (13.2) | 55,628 (13.1) | 18,652 (12.7) |
| 80+ | 23,539 (59.9) | 9,353 (61.6) | - | 351,833 (61.8) | 291,289 (62.9) | 289,245 (62.8) | 246,408 (52.2) | 226,813 (53.4) | 80,524 (54.6) |
| Diabetes, n (%) ^b^ | 13,318 (35.9) | 4,533 (29.9) | - | 181,253 (33.2) | 144,338 (32.1) | 151,189 (32.9) | 156,670 (35.3) | 141,624 (34.9) | 50,836 (34.5) |
| Dementia, n (%) ^b^ | 17,596 (47.5) | 6,634 (43.7) | - | 310,366 (56.8) | 253,529 (56.3) | 273,033 (59.3) | 242,948 (54.7) | 223,304 (55.0) | 84,769 (57.5) |
| Hypertension, n (%) ^b^ | 27,633 (74.6) | 10,907 (71.9) | - | 423,287 (77.4) | 347,784 (77.3) | 356,873 (77.5) | 349,894 (78.8) | 320,994 (79.0) | 115,797 (78.6) |
| Condition information missing, n (%) ^c^ | 2,256 (5.7) | 8 (0.1) | - | 23,218 (4.1) | 13,267 (2.9) | 505 (0.1) | 28,568 (6.1) | 18,819 (4.4) | 106 (0.1) |

a. Age on first included day per person.

b. Prevalence determined by the MDS record with non-missing diagnoses that is most proximal to the first included day per person (among all MDS records within 90 days of this date). Displayed percentages are calculated among the subset with non-missing data.

c. Percent with missing information out of total number of residents.

d. No residents were identified using the 101-day long-stay definition because no lookback data were available to determine prior time in the nursing home.

**Table S3.** Description of all nursing home residents identified using the 101-day long-stay definition and their overlap with the entry-anchored algorithm

|  | All nursing home residents identified using the 101-day long-stay definition between 2013-2020 | Among those in column 1, those who were also found using the entry-anchored algorithm definition | Among those in column 1, those who were not identified in the entry-anchored algorithm definition |
| --- | --- | --- | --- |
| N Residents | 1,897,176 | 1,775,195 | 121,981 |
| N (Goodwin-identified) Days | 1,025,966,142 | 956,435,748 | 69,530,394 |
| % Drug Observable (as identified in the entry-anchored algorithm) | 91.5% | 98.1% | 0% |
| Age, mean (SD) a | 80.0 (12.2) | 80.3 (12.1) | 75.7 (13.4) |
| Age, median (Q1, Q3) a | 82 (72, 89) | 83 (73, 89) | 77 (67, 86) |
| Age, n (%) a |  |  |  |
| <65 | 203,870 (10.8) | 181,273 (10.2) | 22,597 (18.5) |
| 66-69 | 163,261 (8.6) | 148,334 (8.4) | 14,927 (12.2) |
| 70-74 | 187,433 (9.9) | 172,774 (9.7) | 14,659 (12.0) |
| 75-79 | 235,781 (12.4) | 219,951 (12.4) | 15,830 (13.0) |
| 80+ | 1,106,824 (58.3) | 1,052,857 (59.3) | 53,967 (44.2) |
| Diabetes, n (%) b | 636,082 (33.7) | 586,923 (33.2) | 49,159 (41.4) |
| Dementia, n (%) b | 974,883 (51.6) | 923,561 (52.2) | 51,322 (43.2) |
| Hypertension, n (%) b | 1,456,840 (77.1) | 1,364,470 (77.1) | 92,370 (77.7) |
| Condition information missing, n (%) c | 8,356 (0.4) | 5,261 (0.3) | 3,095 (2.5) |
| a. Age on first included day per person.  b. Prevalence determined by the MDS record with non-missing diagnoses that is most proximal to the first included day per person (among all MDS records within 90 days of this date). Displayed percentages are calculated among the subset with non-missing data.  c. Percent with missing information out of total number of residents. | | | |

**Figure S1**. Schematic of overlapping nursing home episodes in the health administrative data


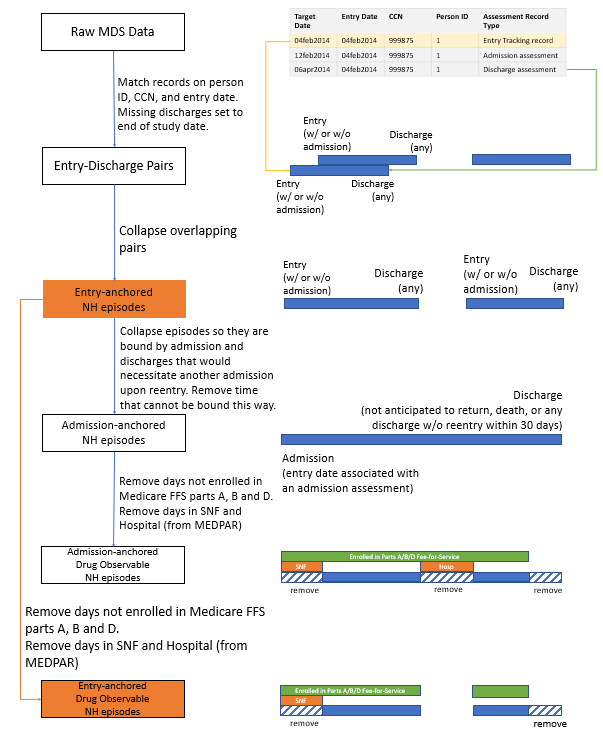

Supplement: Supplementary Materials [file NIHMS2130682-supplement-Supplementary_Materials.docx]
